# Incorporating Amino Acids Composition and Functional Domains for Identifying Bacterial Toxin Proteins

**DOI:** 10.1155/2014/972692

**Published:** 2014-07-07

**Authors:** Min-Gang Su, Chien-Hsun Huang, Tzong-Yi Lee, Yu-Ju Chen, Hsin-Yi Wu

**Affiliations:** ^1^Department of Computer Science and Engineering, Yuan Ze University, Taoyuan 320, Taiwan; ^2^Tao-Yuan Hospital, Ministry of Health & Welfare, Taoyuan 320, Taiwan; ^3^Institute of Chemistry, Academia Sinica, 128 Academia Road, Section 2, Nankang District, Taipei 115, Taiwan

## Abstract

Aside from pathogenesis, bacterial toxins also have been used for medical purpose such as drugs for cancer and immune diseases. Correctly identifying bacterial toxins and their types (endotoxins and exotoxins) has great impact on the cell biology study and therapy development. However, experimental methods for bacterial toxins identification are time-consuming and labor-intensive, implying an urgent need for computational prediction. Thus, we are motivated to develop a method for computational identification of bacterial toxins based on amino acid sequences and functional domain information. In this study, a nonredundant dataset of 167 bacterial toxins including 77 exotoxins and 90 endotoxins is adopted to learn the predictive model by using support vector machines (SVMs). The cross-validation evaluation shows that the SVM models trained with amino acids and dipeptides composition could yield an accuracy of 96.07% and 92.50%, respectively. For discriminating endotoxins from exotoxins, the SVM models trained with amino acids and dipeptides composition have achieved an accuracy of 95.71% and 92.86%, respectively. After incorporating functional domain information, the predictive performance is further improved. The proposed method has been demonstrated to be able to more effectively identify and classify bacterial toxins than the other two features on independent dataset, which may aid in bacterial biomedical development.

## 1. Introduction

The bacterial toxins are capable of causing human diseases. Many pathogenic bacteria produce protein toxins that are important or essential virulent factors [1–3]. Bacterial toxins have many different types which could overcome the defense mechanism of hosts. Typically, they can be classified into endotoxins and exotoxins. The cell-associated toxins are referred to as endotoxins which in most cases reside within the cell wall and are liberated into host tissues upon cell death. Endotoxins are the integral part of the outer membrane of Gram-negative bacteria. Certain proteins and, particularly, the lipopolysaccharides (LPS) comprise the outer layer of this membrane whereas its inner layer is composed of phospholipids and proteins [4]. The exotoxins represent extracellular diffusible toxins which are soluble substances secreted by bacteria in the host tissues [5]. They usually are secreted proteins or polypeptides and act enzymatically or through direct interaction with host cell receptors to stimulate a variety of immune responses, where there can be a site remote from bacterial growth or that of pathogen colonization [1].

The bacterial toxins have been implemented for medical purpose today. For instance, the botulinum toxin, an exotoxin, has been employed in physiatrics, orthopedics, gynecology, pediatrics, neurology, general surgery, plastic surgery, and gastroenterology and also to treat hyperhidrosis and wrinkles in dermatology [6]. Botulinum toxin type B is a safe and effective treatment for upper extremity dystonia in children with cerebral palsy [7]. Cholera toxin (CT) and the heat-labile toxin (LT) have been used as strong mucosal adjuvants in experimental models [8]. Some studies demonstrated that similar bacterial toxins can be effective in the cancer therapy and its feasibility has been cemented by using bacterial toxins to develop drug for cancer, receptor disease, and immune disease [9–12]. With the evolvement of biomedical technology, pathogenic mechanisms of toxin have been revealed gradually and made bacterial toxins potential powerful experimental and clinical tools. To facilitate the bacteria toxin identifications, which are traditionally achieved by costly and time-consuming experimental methods, developing a high throughput method to rapidly and accurately identify bacterial toxins is an urgent need. Besides, endotoxins and exotoxins have different roles and mechanism in the host, thus, classification of bacterial toxins provide vital clues in understanding basic cell biology and further development of toxoid based therapeutics. To address the above issues, by using support vector machines (SVM) and dipeptides composition, BTXpred has proposed and achieved an accuracy of 96.07 and 92.50% for discriminating bacteria toxins and nontoxins, respectively. They also obtained 95.71 and 92.86% of accuracy in classifying endotoxins and exotoxins, respectively [13]. Yang et al. achieved higher MCC in the same dataset by using increment of diversity and SVM [14]. Based on the concept of pseudo amino acid composition, combined with the methods of approximate entropy and IB1 algorithm, Song has demonstrated higher accuracy than the previous two methods [15]. In this study, we attempted to not only use amino acid and dipeptide composition but also develop a method containing functional domain mapping for identification of bacterial toxins and classification of bacterial toxins (endotoxins and exotoxins).

## 2. Material and Methods

**Figure 1:**
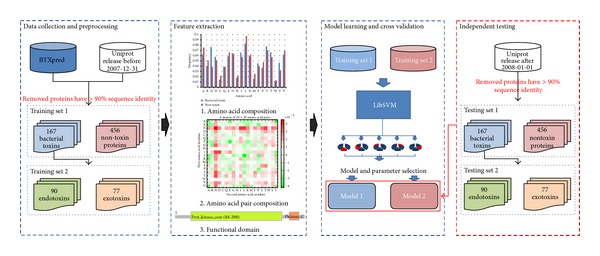
Systematic workflow. It consists of the following steps: data collection and preprocessing, feature extraction, model learning and cross validation, and independent testing.

Figure 1 presents the systematic workflow of the proposed method. It consists of the following steps: data collection and preprocessing, feature extraction, model learning and cross validation, and independent testing. The details of each process were described as follows.

## 3. Data Collection and Preprocessing

The training dataset used in this study was obtained from BTXpred [13] which was originally collected from UniProt database [16]. In BTXpred, nontoxin protein sequences were also obtained from UniProt by combined search using SRS. The retrieved data were checked in order to eliminate toxin proteins [17]. After removing sequences that have more than 90% sequence identity, the nonredundant dataset in BTXpred consists of 150 bacterial toxins that have 77 exotoxins and 73 endotoxins. The exotoxins were further classified based on their molecular targets. Here, we have removed proteins annotated as “Obsolete” in UniProt and added additional data released on UniProt by “December 31, 2007”. The final nonredundant dataset consists of 456 nontoxin proteins and 167 bacterial toxins containing 90 endotoxins and 77 exotoxins for model training.

For independent testing dataset, we collected another bacterial toxin data based on previous work [13] including the updated data released after “January 01, 2008” on UniProt. Homologous sequences from the testing data were removed by using CD-HIT [18]. The final testing dataset included 810 nontoxin protein and 271 bacterial toxin proteins which contain 162 endotoxins and 109 exotoxins for independent testing. There also have 90% sequence similarity between training dataset and testing dataset.

### 3.1. Feature Extraction

#### 3.1.1. Compositions of Amino Acids and Amino Acid Dipeptide

Each protein sequence in the dataset was represented using a vector {*x*_*i*_, *i* = 1,…, *n*} labeled according to its corresponding protein group (e.g., bacterial toxins or nontoxin proteins). The vector *x*_*i*_ has 20 elements for the amino acid composition and 400 elements for the amino acid dipeptide composition. For amino acid composition, the 20 elements specified the numbers of occurrences of 20 amino acids normalized with the total number of residues in the protein. On the other hand, for amino acid dipeptide composition, the 400 elements specified the numbers of occurrences of 400 amino acid dipeptides normalized with the total number of dipeptides in the protein.

#### 3.1.2. Information of Functional Domains

Previous works on protein prediction have exhibited the ability of distinguishable domain regions in the classification of proteins [19, 20]. In this work, domain information was investigated as a feature for classifying bacterial toxins from nontoxin proteins. To investigate the preference of functional domains in bacterial toxins, this study referred to the annotations in InterPro [21]. InterPro is an integrated resource, which was developed initially as a means of rationalizing the complementary efforts of the PROSITE [22], PRINTS [23], Pfam [24], and ProDom [25] databases, for providing protein “signatures” such as protein families, domains, and functional sites. The domain information of each bacterial toxin in the training data was collected by referring to its corresponding InterPro ID in the UniProt database. The collected domains were then analyzed in order to identify the most distinguishable domains in bacterial toxin. In this work, functional domains presenting in more than five bacterial toxins were considered as significant domains.

### 3.2. Model Learning and Cross Validation

A support vector machine (SVM) was applied to generate computational models that incorporate the encoded features such as amino acids, accessible surface area, and secondary structure. Based on binary classification, the concept of SVM is to map the input samples into a higher dimensional space using a kernel function and then to find a hyperplane that discriminates between the two classes with maximal margin and minimal error. A public SVM library, LibSVM [26], was used to train the predictive model with positive and negative training sets, which were encoded with reference to various training features. The radial basis function (RBF) *K*(*S*_*i*_, *S*_*j*_) = exp⁡(−*γ*||*S*_*i*_−*S*_*j*_||^2^)  was selected as the kernel function of SVM. Moreover, two SVM parameters, cost and gamma value, were optimized to maximize predictive accuracy.

Cross-validation evaluation is important for the application of a predictor [27]. The predictive performance of the constructed models was evaluated by performing *k*-fold cross validation. The training data was divided into *k* groups by splitting each dataset into *k* approximately equal sized subgroups. In one round of cross validation, a subgroup was regarded as the test set, and the remaining *k*-1 subgroups were regarded as the training set. The cross-validation process was repeated *k* rounds, with each of the *k* subgroups used as the test set in turn. Then, the *k* results were combined to produce a single estimation. The advantage of *k*-fold cross validation was that all original data were regarded as both training set and test set, and each data was used for testing exactly once [28]. In this study, *k* was set to five. The following measures of predictive performance of the trained models were defined:

(1) $$\begin{array}{c} \text{Sensitivity}\left(\text{Sn}\right)=\frac{\text{TP}}{\text{TP}+\text{FN}},\\ \text{Specificity}\left(\text{Sp}\right)=\frac{\text{TN}}{\text{TN}+\text{FP}},\\ \text{Accuracy}\left(\text{Acc}\right)=\frac{\text{TP}+\text{TN}}{\text{TP}+\text{FN}+\text{TN}+\text{FP}}, \end{array}$$

where TP, TN, FP, and FN represented the numbers of true positives, true negatives, false positives, and false negatives, respectively. Additionally, the parameters of the predictive models, window length, cost, and gamma value of the SVM models were optimized to maximize predictive accuracy. Finally, the parameters that yielded the highest accuracy were employed to construct predictive models for independent testing.

### 3.3. Independent Testing

In order to further evaluate the trained models for identify bacterial toxin, an independent test set was obtained as described previously, resulting in 271 positive data and 810 negative data. In addition, this work also investigated the ability of the predictive model to identify bacterial toxin subtype: “exotoxin” and “endotoxin.”

## 4. Results and Discussion

### 4.1. Investigation of Amino Acid Composition in Bacterial Toxins

**Figure 2:**
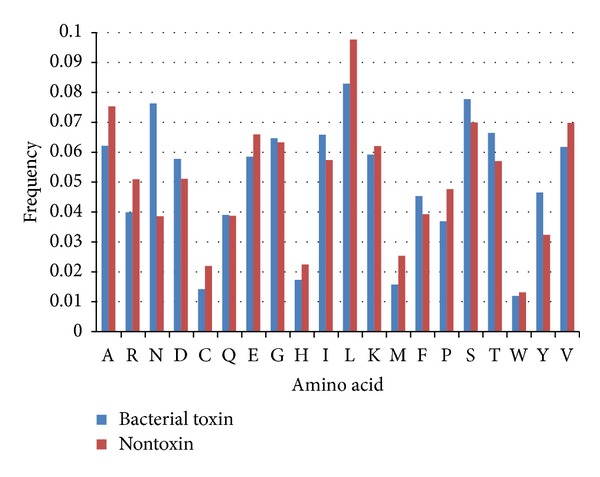
Percent composition of 20 amino acids between positive data (bacterial toxin proteins) and negative data (nontoxin proteins).

**Figure 3:**
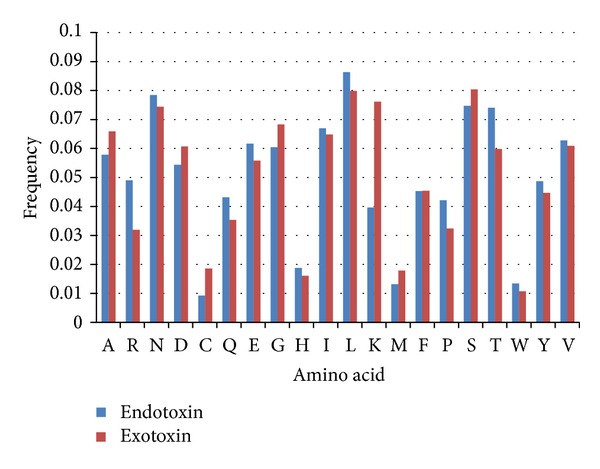
Percent composition of 20 amino acids between endotoxin and exotoxin.

The difference between bacterial toxin and nontoxin proteins was analyzed in terms of its amino acid composition and the result was shown in Figure 2. To determine the differentially presented amino acid, the occurrence of each amino acid except for N which is of the highest frequency (0.03772) was averaged. After adding the standard deviation, 0.004225, to the average (0.00767), 0.011895 was considered to be the threshold. It can be observed that bacterial toxins are significantly distinguishable from nontoxin proteins at the amino acid composition level. For instance, alanine (A, 0.01317), asparagine (N, 0.03772), leucine (L, 0.01475), and tyrosine (Y, 0.01416) residues all exhibit a remarkable difference between bacterial toxin and nontoxin proteins. Asparagine (N) is the most significantly distinguishable among all residues. In order to examine the effectiveness of amino acid composition in identifying baterial toxins, an SVM model was trained using a 20-dimensional vector consisting of the composition scores for twenty amino acids. The amino acid composition-based model was evaluated by means of five-fold cross validation. As shown in Table 1, the model achieved sensitivity of 92.81%, specificity of 99.56%, and accuracy of 97.75%. Amino acid composition comparison between endotoxin and exotoxin was also performed and shown in Figure 3. The occurrence of each amino acid except for most distinguishable residue, K, was used to obtain an average (0.006347). After adjusting the average by sytandard deviation (0.004226), frequency larger than 0.010573 was considered to be differential. Arginine (R, 0.017043), lysine (K, 0.03654), and threonine (T, 0.014236) residues were found to have differential frequency between endotoxin and exotoxin proteins. Similarly, SVM model was trained using a 20-dimensional vector consisting of the composition scores for twenty amino acids and evaluated by means of five-fold cross validation. As shown in Table 2, the model achieved sensitivity of 93%, specificity of 93.93%, and accuracy of 94.02%.

### 4.2. Investigation of Amino Acid Dipeptide Composition in Bacterial Toxins

Protein dipeptide composition has been widely used in the proteins identification. Previous studies have reported that dipeptide composition-based methods can yield a better performance compared to amino acid composition-based methods [29, 30]. To investigate this claim in terms of identifying bacterial toxins, an SVM model was trained by using amino acid dipeptide composition as features. Firstly, the composition of all possible amino acid pairs was calculated in baterial toxins and nontoxin proteins, leading to the fact that each protein sequence can be encoded as a 400-dimensional vector consisting of the composition scores for 20 × 20 amino acid pairs. Using the resulting 400-dimensional dipeptide vectors, an SVM model was trained and is evaluated by means of five-fold cross validation.

**Figure 4:**
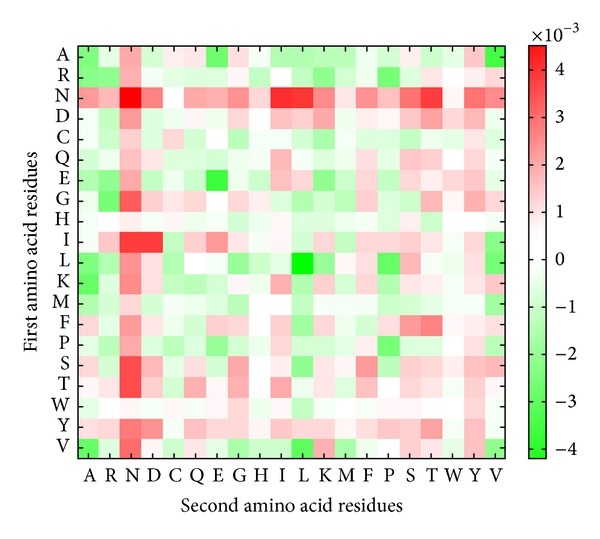
Probability difference of 20 × 20 amino acid pairs between bacterial toxin proteins and nontoxin proteins. The amino acid pair with red box indicates an overrepresentation in bacterial toxin proteins; on the other hand, green box means an underrepresentation.

**Figure 5:**
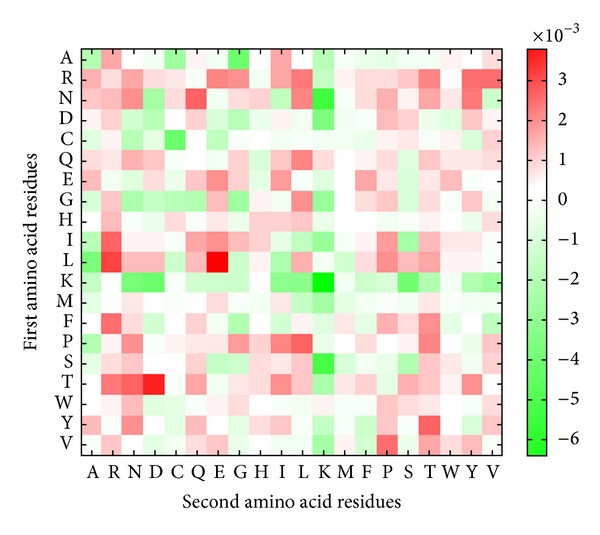
Probability difference of 20 × 20 amino acid pairs between endotoxin and exotoxin. The amino acid pair with red box indicates an overrepresentation in endotoxin; green box means an overrepresentation in exotoxin.

The performance of dipeptide composition-based model for identifying bacterial toxin has sensitivity of 87.42%, specificity of 96.71%, and accuracy of 94.06% (as shown in Table 1). It can be observed that the amino acid composition-based method yields higher accuracy in identifying bacterial toxins. It may be due to the short sequence length of toxins as it is difficult to obtain significant number of dipeptides for small proteins [13]. The amino acid dipeptide composition of bacterial toxins and nontoxin proteins is further analyzed by selecting statistically significant dipeptides among the 400 amino acid pairs.  Figure 4 shows the probability difference of 400 amino acid pairs between bacterial toxins and nontoxin proteins. In the 20 × 20 matrix, amino acid pairs marked in red indicates overrepresentation in bacterial toxins, while amino acid pairs marked in green indicates underrepresentation. As illustrated in Figure 4, NN pairs are overrepresented in bacterial toxins as well as N residues paired with I, L, and T. Similarly, the amino acid dipeptide composition-based method also yields lower accuracy in classifying exotoxin and endotoxin as compared to amino acid composition-based methods. The model achieved sensitivity of 92.22%, specificity of 85.71%, and accuracy of 89.22%, as shown in Table 2.  Figure 5 portraits the probability difference of 400 amino acid pairs between endotoxin and exotoxin proteins. Amino acid pairs marked in red indicates overrepresentation in endotoxin, while amino acid pairs marked in green indicates overrepresentation in exotoxin. It can be observed that LE and TD pairs are overrepresented in endotoxin, while SK, KK, and NK pairs are overrepresented in exotoxin.

### 4.3. Investigation of Functional Domain Information in Bacterial Toxins

In order to analyze functional domain information in bacterial toxin, the experimentally verified domains of each baterial toxin in the training data were collected by referring to the “InterPro” field in UniProt, resulting in a total of 100 functional domains. Since some toxin domains were filtered out while applying 6 baterial toxins, here, in order to capture the representative functional domains in bacterial toxins, functional domains that are present in more than 5 bacterial toxins were selected as distinguishable domains. Accordingly, a total of 40 functional domains were obtained as shown in Table 3. It is observed that the most distinguishable functional domain is the “Endotoxin_N” with InterPro ID: IPR005639 which is composed of 84 bacterial toxins. Other distinguishable functional domains that comprise more 60 bacterial toxin proteins are the Galactose-bd-like, Endotoxin_C, Endotoxin_cen_dom, and Endotoxin_cen_dom_subgrl with InterPro ID IPR008979, IPR005638, IPR001178, and IPR015790, respectively. It is noticeable that these discernible functional domains all exist in endotoxins, implying that most of the endotoxins have similar functions while exotoxins still can be divided into more subcategories according to different functions. Inspired by this observation, we further evaluate the performance of using the selected distinguishable domains. An SVM model was trained using a 15-dimensional vector consisting of the 40 distinguishable domains represented by a binary score: 1 if present and 0 otherwise. As shown in Table 1, the domain-based model for indentifying bacterial toxin proteins yielded sensitivity of 100%, specificity of 94.73%, and accuracy of 96.15%. We found out that bacterial toxins can be identified with best sensitivity by domain information compared to the other two prediction models. The result also suggested that 40 functional domains have covered that in all bacterial toxin proteins. On the other hand, the high specificity yielded by the model signifies that the selected functional domains are meaningful since they do not exist in most of the non-toxin proteins. Inspired by the result, the domain-based model was further applied on classifying endotoxins and exotoxins. As shown in Table 2, the prediction has accomplished sensitivity of 100%, specificity of 82.2%, and accuracy of 90.42%. High sensitivity indicated that domain information is capable of correctly identifying endotoxin owing to the fact that most of distinguishable functional domains are located in endotoxin. However, specificity yielded by the model points to the fact that some selected functional domains both exist in endotoxin and exotoxin. Domain information could play a complementary role for prediction of class of baterial toxins.

### 4.4. Independent Testing

The feasibility of this method was further evaluated by using an independent dataset composed of collected bacterial toxin proteins with two subtypes (edotoxin and exotoxin) and nontoxin proteins as described above. The independent data was first tested on models of bacterial toxin and nontoxin trained on each feature as shown in Table 4. The amino acid composition-based model has sensitivity of 30.99%, specificity of 93.95%, and accuracy of 78.17%. And sensitivity of 27.31%, specificity of 88.89%, and accuracy of 73.45% were gained by using dipeptide composition-based model. Obviously, both two models yielded a much lower performance as compared to the models based on functional domain that have sensitivity of 99.63%, specificity of 93.95%, and accuracy of 95.37%. The independent data was further tested for classifying exotoxin and endotoxin by each feature. As presented in Table 5, the functional domain-based model still yielded better performance as compared to the other two models by having sensitivity of 94.44%, specificity of 73.39%, and accuracy of 85.98%. The amino acid composition-based model performs with sensitivity of 0%, specificity of 94.49%, and accuracy of 38.01%. Amino acid composition-based model performs with sensitivity of 11.72%, specificity of 89%, and accuracy of 43.17%.

## 5. Conclusion

Bacterial toxins have wide-range affection on not only host defense and immune response but also therapeutics interventions. It is conceivable that how to identify bacteria toxins and its subtypes in a rapid and accurate way by taking advantage of computing science information is significant and helpful. Many research works have been working in this field to facilitate the understanding of bacterial toxicology and development of therapeutic drugs and vaccines. From the results of these studies, machine learning methods are suitable for toxin proteins prediction due to the sequencing of most genomes of 296 bacteria, leading to a high accuracy.

Here we have proposed a different computational method to identify bacterial toxin proteins on the basis of amino acid sequence and functional domain information of a protein. Since shorter toxin protein sequences are commonly found and the different types of toxins having different specific functionality, functional domains information are more suitable for identifying the type of bacterial toxins as compared to amino acids composition information in protein sequence. From independent test results, the functional domain information provided a more stable performance for identification of bacterial toxins. It is speculated that even if the amino acid composition is different, the specific function domains are conserved. This work shows that the* in silico* identification could be a feasible mean for conducting preliminary analyses as well as significantly reducing the number of potential targets that require further* in vivo* or* in vitro* experimental confirmation. It is invisible that the functional domain based model may have potential for predicting toxin proteins produced by other species.

## Figures and Tables

**Table 1:** Five-fold cross-validation performance of basic features in prediction of bacterial toxins.

Features	Sensitivity	Specificity	Accuracy
Amino acid composition	92.81%	99.56%	97.75%
Dipeptide composition	87.42%	96.71%	94.06%
Functional domain	100.0%	94.73%	96.15%

**Table 2:** Five-fold cross-validation performance of basic features in discrimination of endotoxins and exotoxins.

Features	Sensitivity	Specificity	Accuracy
Amino acid composition	93%	93.93%	94.02%
Dipeptide composition	92.22%	85.71%	89.22%
Functional domain	100.0%	82.2%	90.42%

**Table 3:** Statistics of InterPro functional annotations in 167 bacterial toxin proteins. InterPro classifies sequences at superfamily, family, and subfamily levels and annotates of the occurrence of functional domains, repeats, and important sites. The annotations which occur in more than five bacterial toxins are presented with the information of InterPro ID, description, and bacterial toxin proteins.

InterPro ID	Description	Number of bacterial toxin proteins
IPR005639	Endotoxin_N	84
IPR008979	Galactose-bd-like	83
IPR005638	Endotoxin_C	82
IPR001178	Endotoxin_cen_dom	75
IPR015790	Endotoxin_cen_dom_subgr1	68
IPR006123	Toxin_b-grasp_Staph/Strep	15
IPR006126	Staph/Strept_toxin_CS	15
IPR006173	Staph_tox_OB	15
IPR008992	Enterotoxin_bac	15
IPR016091	SuperAg_toxin_C	15
IPR006177	Toxin_bac	14
IPR013307	Superantigen_bac	14
IPR003995	RTX_toxin_determinant-A	13
IPR011049	Serralysin-like_metalloprot_C	13
IPR018504	RTX_N	13
IPR001343	Hemolysn_Ca-bd	12
IPR001340	Leukocidin/haemolysin_toxin	11
IPR001489	Heat-stable_enterotox_STa	11
IPR008985	ConA-like_lec_gl_sf	11
IPR013320	ConA-like_subgrp	11
IPR018511	Hemolysin-typ_Ca-bd_CS	11
IPR019806	Heat-stable_enterotox_CS	11
IPR000395	Neurotox_Zn_protease	9
IPR001869	Thiol_cytolysin	9
IPR011065	Kunitz_inhibitor_ST1-like	9
IPR012500	Toxin_trans	9
IPR012928	Toxin_rcpt-bd_N	9
IPR013104	Toxin_rcpt-bd_C	9
IPR013550	RTX_C	9
IPR015214	Endotoxin_cen_dom_subgr2	8
IPR001615	Endotoxin_CytB	7
IPR003963	Bi-component_toxin_staph	7
IPR003842	Vacuolating_cytotoxin	6
IPR005015	Thermostable_hemolysn_vibrio	6
IPR005546	Auto_transptbeta	6
IPR008947	PLipase_C/P1_nuclease	6
IPR001024	PLAT/LH2_dom	5
IPR001144	Enterotoxin_A	5
IPR001531	PLipaseC_domain	5
IPR008976	Lipase_LipOase	5

**Table 4:** Predictive performance of basic features for distinghishing bacterial toxins and nontoxins on an independent testing data.

Features	Sensitivity	Specificity	Accuracy
Amino acid composition	30.99%	93.95%	78.17%
Dipeptide composition	27.31%	88.89%	73.45%
Functional domain	99.63%	93.95%	95.37%

**Table 5:** Predictive performance of basic features for distinghishing endo- and exotoxins on an independent testing data.

Features	Sensitivity	Specificity	Accuracy
Amino acid composition	0%	94.49%	38.01%
Dipeptide composition	11.72%	89%	43.17%
Functional domain	94.44%	73.39%	85.98%

## References

[1] Alouf JE, Popoff MR (2006). *The Comprehensive Sourcebook of Bacterial Protein Toxins*.

[2] Bourne HR (1990). ADP-Ribosylating Toxins and G Proteins. Insights into Signal Transduction. Joel Moss and Martha Vaughan, Eds. American Society for Microbiology, Washington, DC, 1990. xviii, 567 pp., illus. *79; to ASM members*, 69. *Science*.

[3] Montecucco C, Papini E, Schiavo G (1994). Bacterial protein toxins penetrate cells via a four-step mechanism. *FEBS Letters*.

[4] Reitschel ET, Kirikae T, Shade FU (1994). Bacterial endotoxin: molecular relationships of structure to activity and function. *FASEB Journal*.

[5] Prescott LM, Harley JP, Klein DA (1993). Symbiotic associations: parasitism, pathogenicity and resistance. *Microbiology*.

[6] Tamura BM, Chang B (2003). Botulinum toxin: application into acupuncture points for migraine. *Dermatologic Surgery*.

[7] Sanger TD, Kukke SN, Sherman-Levine S (2007). Botulinum toxin type B improves the speed of reaching in children with cerebral palsy and arm dystonia: an open-label, dose-escalation pilot study. *Journal of Child Neurology*.

[8] Sun JB, Czerkinsky C, Holmgren J (2010). Mucosally induced immunological tolerance, regulatory T cells and the adjuvant effect by cholera toxin B subunit. *Scandinavian Journal of Immunology*.

[9] Ansiaux R, Gallez B (2007). Use of botulinum toxins in cancer therapy. *Expert Opinion on Investigational Drugs*.

[10] Cheong I, Huang X, Bettegowda C (2006). A bacterial protein enhances the release and efficacy of liposomal cancer drugs. *Science*.

[11] Nuyts S, Theys J, Landuyt W, van Mellaert L, Lambin P, Anné J (2001). Increasing specificity of anti-tumor therapy: cytotoxic protein delivery by non-pathogenic clostridia under regulation of radio-induced promoters. *Anticancer Research*.

[12] Patyar S, Joshi R, Byrav DSP, Prakash A, Medhi B, Das BK (2010). Bacteria in cancer therapy: a novel experimental strategy. *Journal of Biomedical Science*.

[13] Saha S, Raghava GPS (2007). BTXpred: prediction of bacterial toxins. *In Silico Biology*.

[14] Yang L, Li QZ, Zuo YC, Li T (2009). Prediction of animal toxins using amino acid composition and support vector machine. *Inner Mongolia University*.

[15] Song C (2011). Prediction of bacterial toxins by an improved feature extraction and IB1 algorithm fusion. *African Journal of Microbiology Research*.

[16] Apweiler R, Bairoch A, Wu CH (2004). UniProt: the universal protein knowledgebase. *Nucleic Acids Research*.

[17] Saha S, Raghava GP (2007). Prediction of neurotoxins based on their function and source. *In Silico Biology*.

[18] Huang Y, Niu B, Gao Y, Fu L, Li W (2010). CD-HIT Suite: a web server for clustering and comparing biological sequences. *Bioinformatics*.

[19] Wang L, Huang C, Yang JY (2010). Predicting siRNA potency with random forests and support vector machines. *BMC Genomics*.

[20] Hsu JB-K, Bretaña NA, Lee T-Y, Huang H-D (2011). Incorporating evolutionary information and functional domains for identifying RNA splicing factors in humans. *PLoS ONE*.

[21] Hunter S, Apweiler R, Attwood TK (2009). InterPro: the integrative protein signature database. *Nucleic Acids Research*.

[22] Bairoch A (1991). PROSITE: a dictionary of sites and patterns in proteins. *Nucleic Acids Research*.

[23] Attwood TK, Beck ME, Bleasby AJ, Parry-Smith DJ (1994). PRINTS: a database of protein motif fingerprints. *Nucleic Acids Research*.

[24] Sonnhammer EL, Eddy SR, Durbin R (1997). Pfam: a comprehensive database of protein domain families based on seed alignments. *Proteins*.

[25] Corpet F, Gouzy J, Kahn D (1998). The ProDom database of protein domain families. *Nucleic Acids Research*.

[26] Chang C-C, Lin C-J LIBSVM: a library for support vector machines. http://www.csie.ntu.edu.tw/~cjlin/libsvm.

[27] Chou KC, Shen HB (2007). Recent progress in protein subcellular location prediction. *Analytical Biochemistry*.

[28] Ron K A study of cross-validation and bootstrap for accuracy estimation and model selection.

[29] Bhasin M, Raghava GPS (2004). Classification of nuclear receptors based on amino acid composition and dipeptide composition. *The Journal of Biological Chemistry*.

[30] Panwar B, Raghava GP (2010). Prediction and classification of aminoacyl tRNA synthetases using PROSITE domains. *BMC Genomics*.

